# SISE, free LabView-based software for ion flux measurements

**DOI:** 10.1186/s13007-025-01448-8

**Published:** 2025-11-18

**Authors:** Namrah Ahmad, Krishani Tennakoon, Rainer Hedrich, Shouguang Huang, M. Rob G. Roelfsema

**Affiliations:** 1 Molecular Plant Physiology and Biophysics, Julius-von-Sachs Institute for Biosciences, Biocenter, Würzburg University, Julius-von-Sachs-Platz 2, D-97082 Würzburg, Germany; 2 Faculty of Synthetic Biology, Shenzhen University of Advanced Technology, Shenzhen, 518055 China; 3 Key Laboratory of Quantitative Synthetic Biology, Shenzhen Institute of Synthetic Biology, Shenzhen Institutes of Advanced Technology, Chinese Academy of Sciences, Shenzhen, 518055 China

## Abstract

Plant growth and development strongly depend on the uptake of soil minerals and their distribution within plants. Various electrophysiological techniques have been developed to study these ion transport processes and the role of ions in signal transduction pathways. An important non-invasive method is provided by Scanning Ion-Selective Electrodes (SISE), which are used to detect ion fluxes. These SISE-measurements depend on software that coordinates the continuous electrode movement between two positions, as well as data collection and analysis. We developed two LabView-based programs; the SISE-Monitor and SISE-Analyser that enable ion flux recordings and their analysis, respectively. These applications are freely available, both as windows-executable files that enable routine measurements, as well as the LabView source code that allows insights into the routines used for measurement and analysis. Moreover, the source code can be used to develop new functions, such as the combined measurement of extracellular ion fluxes with SISE and cellular ion concentrations with fluorescent dyes, or proteins.

**Supplementary Information:**

The online version contains supplementary material available at 10.1186/s13007-025-01448-8.

## Background

Ion transport plays a major role in the growth and development of plants. Nutrients are taken up from the soil in their aqueous ionic form and transported within the plant body, via the xylem and phloem network. At their destination, these nutrients enter the cells of growing tissues, where they support osmotically driven cell expansion, as well as a multitude of specific functions. Moreover, Ca^2+^ ions are involved in various signaling processes in plants [1] and strongly linked to H^+^ uptake, or release [2]. Because of the central role of ion transport in the physiology of plants [3], a variety of electrophysiological techniques have been developed to study these processes.

In the 1990s, techniques were established to study plant ion fluxes with extracellular ion-selective electrodes. These minimal invasive techniques enabled measurements of ion transport with intact tissues and single cells, such as leaves of aquatic plants [4], roots [5, 6] and pollen tubes [7, 8]. For large tissues, like leaves of the aquatic plant *Potamogeton lucens*, two miniature pH electrodes (diameter 1,5 mm) could be used at a fixed distance of 5 mm, to evaluate H^+^ fluxes in and out of leaves [4].

Studies on smaller tissues, or single cells, required smaller ion-selective electrodes, which can be constructed from pulled glass capillaries, plugged with liquid membranes. The tip size of these electrodes is approximately 10 μm, which enables measurements on normal-sized plant cells [7]. However, the voltage of these electrodes tends to drift, which may be due to loss of components from the ion-selective cocktail in the tips of these electrodes, or an imperfect seal between the cocktail and the silanized glass capillary [9, 10]. Consequently, it is difficult to use two liquid membrane electrodes positioned at a fixed distance, as explained above for *P. lucens*. Instead, a single ion-selective electrode is used, which moves perpetually between two positions. The use of such “self-referencing” scanning electrodes has become established and has been described in several method papers [11–14].

Even though all studies with self-referencing ion-selective electrodes are based on diffusion in unstirred layers of solution, different approaches have been developed to calculate the ion fluxes [13, 15]. Whereas the group of Smith et al. (1994) used Fick’s law of diffusion [15] to determine the ion fluxes, Newman (2001) developed an approach that is based on the difference in chemical potential between the two positions [13].

Despite using the same technical approach, the ion flux measurements methods have obtained various names, such as ion-selective vibrating-microelectrode system [6], Scanning Ion-selective Electrode Technique (SIET) [16], Microelectrode Ion Flux Estimation (MIFE) [14], or Non-invasive Micro-test System (NMT) [17]. To some extend these names are protected and we therefore will use the description Scanning Ion-Selective Electrode (SISE), or just “ion flux measurement”.

Central to ion flux measurements is a software that controls the electrode movement and collects data from the amplifier that is connected to the ion-selective electrode. In the period from 2013 to 2015, we encountered limitations with commercial software and started to develop two LabView-based programs to conduct and analyse ion flux measurements. LabView provides a graphical environment to program “test and measurement” software that is widely used by engineers and scientists. A community edition is provided that can be downloaded for free and used for non-commercial projects (https://www.ni.com/en/shop/labview/select-edition/labview-community-edition.html). The software is especially designed to support data acquisition and control measuring devices (https://www.ni.com/en/shop/labview.html) and is thus very well suited to control electrode movements and data collection during ion flux measurements.

The SISE-Monitor offers a user-friendly approach to register time-dependent changes in the voltage signal of the ion-selective electrode, which enables the experimenter to judge the validity of measurements. A second program, the SISE-Analyser, allows the re-evaluation of time-dependent voltage changes and calculate the ion fluxes, offline. These options were not available in the commercial software that we obtained in 2013 and have been crucial for ion flux measurements that were conducted in several research projects since 2018 [18–21]. The LabView source code is now made publicly available in the SISE-Virtual-Instruments(VI)-files folder through GitHub (https://github.com/Rob-Roelfsema/SISE-Software-April2025), which enables insights into the routines used in the SISE programs and allow new features to be implicated, using the LabView programing environment, which is freely available for non-commercial purposes as a “community edition”.

## Results

The SISE-software is separated in two applications; (i) the SISE-Monitor that supports the measurements in real time and (ii) the SISE-Analyser to conduct offline analysis and calculate ion-fluxes. The basic properties of both applications will be described below. A point-by-point manual that explains how the software is installed and used, as well as a protocol to test the SISE measurement setup are provided in the supplementary information.

### SISE-monitor

Two versions of the SISE-Monitor will be provided via GitHub (https://github.com/Rob-Roelfsema/SISE-Software-April2025). The SISE-Monitor100 and 110 are a fully active applications that can be used in combination with the Patchstar micromanipulator (Scientifica, Uckfield, UK) and various analog-to-digital converters of NI (National Instruments Corp., Austin, TX, USA). Both versions differ in the timing of the experiments, whereas the duration of experiments will be slightly longer, as the time specified at the start of the measurement in version *100, timing is precise in version *110. However, version *110 has not been tested as rigorously, as in version *100 and we therefore provide both versions.

In addition, demonstration versions of the SISE-Monitor are supplied (SISE-monitor100 and 110-demo), which provide an impression of the abilities of the program, without having to invest time and money into hardware components. These versions can also be used to generate synthetic data sets, which can be used to evaluate the functions of the SISE-Analyser program (see below).

All programs are available as compiled programs (*.exe), and as “virtual instrument” (*.vi) files. The *.exe files run on a windows computer, in combination with a LabView runtime engine, which can be downloaded from the NI-website for free. The *.vi files can be opened in LabView and will allow modification of the program. The description below will apply to the normal SISE-Monitor100 and 110 programs, but most of it is also valid for the demo versions.

**Fig. 1:**
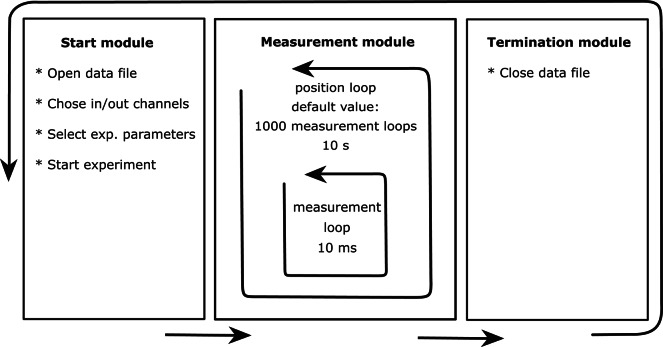
Schematic overview of the main modules of the SISE-Monitor. In the start module, a data file is generated, the input channels are chosen, and experimental parameters are selected, before the program is started. In the measurement module, a fast “measurement loop” is active that stores data at an interval of exactly 10 ms in the SISE-Monitor110 (in version 100 some additional time will be added to the 10 ms measurement period). At default conditions, the measurement loop is executed 1000 times within the “position loop” and thus adds up to a total time of 10 s at one position. In the next “position loop” the electrode moves to the other position and again 1000 datapoints are acquired at default conditions. The data file is closed in the termination module and the next measurement can be started

The monitor program consists of three main modules (Fig. 1), which are meant to be executed sequentially. In the first module, information needs to be entered about the hardware, data file and experimental conditions. The measurements are carried out in the second module and data storage is finalized in the last module.

### Start-up module

**Fig. 2:**
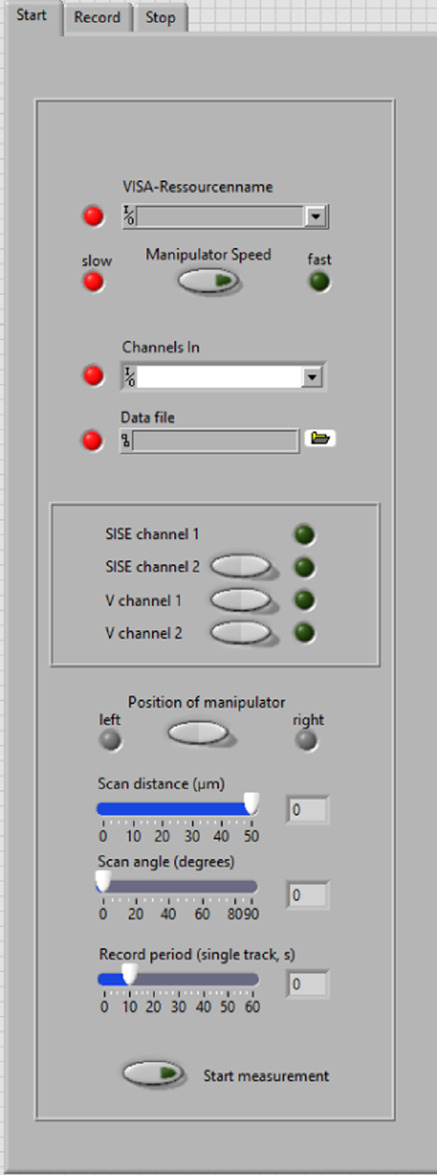
Start window of the SISE-monitor. *Upper part*: Before the SISE-monitor can be started several parameters have to be entered. In the upper “VISA-Resource name” window, the COM-port to which the micromanipulator is connected is chosen. The virtual LED next to the window will turn green once a parameter is correctly entered. The manipulator can be switched between a fast and slow mode with the “Manipulator Speed” switch. The measurement can only be started with the manipulator in the slow speed mode. In the “Channel in” window, four channels of the NI-interface need to be selected, corresponding to the SISE channel 1 and 2 and Voltage channel 1 and 2, respectively. In the window below, a data file name must be provided. *Middle part.* The switches allow selection of the data channels that will be recorded in addition to SISE channel 1. *Lower part*: A switch is provided to enable measurements with a manipulator positioned on the right, or left side, of the tissue of interest. In addition, three sliders are available to set the scan distance of the ion-selective electrode, the angle at which the electrode moves (0 degrees is horizontal) and the period of measurement at one position. The measurement can be started, once all virtual LEDs are green, by pushing the switch at the bottom of the window

Before the SISE-monitor can be started, information needs to be provided about the hardware and experimental conditions (Fig. 2). First, the communication (COM) port of the Patchstar micromanipulator (Scientifica) needs to be selected. If the correct port is chosen, the virtual LED next to the COM-port window, will turn green. Below the COM-port window, the speed of the manipulator can be changed from a fast mode, which is useful to position the electrode, to the slow mode that is required to start the SISE recording. Next, four input channels of the analog-to-digital (A/D) converter need to be selected (refer to the manual for further details) and a data file name must be provided.

The program can be used with a variety of NI A/D converters, but it is advisable to use high-end A/D converters, to minimize the noise level and thereby enhance the resolution of the measurements. The first chosen analog input channel (in most cases AI0) should be connected to the first ion-selective electrode amplifier. A second SISE channel can be selected, by pushing the virtual switch (“SISE channel 2“), just as two additional voltage channels. These data source channels will be assigned as; AI1, AI2 and AI3, to further analog input channels. In total the program thus can store data of 4 channels; 2 SISE channels and 2 additional voltage channels. 

Measurements can be conducted on the left, or right side, of the tissue that is studied. The position of the manipulator must be provided, to ensure that first movement of the electrode is away from the tissue (or cell). Finally, 3 parameters need to be selected that define the distance, the angle, and the interval by which the micromanipulator moves.

 Once all virtual LEDs have turned green, the measurements can be started.

### Measurement module

**Fig. 3:**
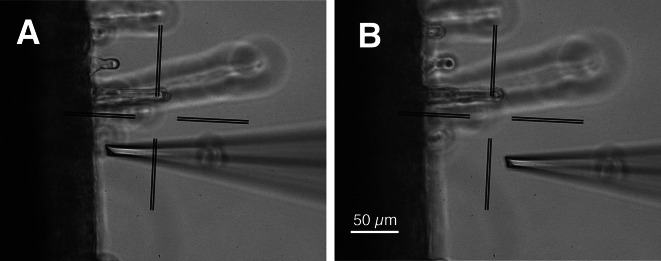
Ion flux measurement on a root of a barley seedling. **A** A K^+^-selective electrode with its tip at position 0, close an epidermal cell in the root hair zone. **B** The tip of the electrode moved 50 μm sidewards, to position 1

The movements of the micromanipulator are controlled by “Virtual Instrument Software Architecture” (VISA) comments, which can be downloaded, as a NI-VISA file, from the NI website (https://www.ni.com). Before the start of the experiment, the electrode tip must be moved manually to position 0, close to the tissue of interest (the distance should be documented). Upon start of the experiment, the electrode will remain at position 0 during the first measurement and only will move away from the tissue, to position 1, during the next measurement (Fig. 3).

By default, the electrodes move horizontally (angle of 0°), over a distance of 50 μm, every 10 s, but these values can be altered before the start of the experiment. A measurement on a root of a barley seedling is shown in Fig. 3, in which the electrode is close to the root surface in position 0 (Fig. 3A), while its tip moves 50 μm to the right at position 1 in Fig. 3B.

### SISE channel data collection

**Fig. 4:**
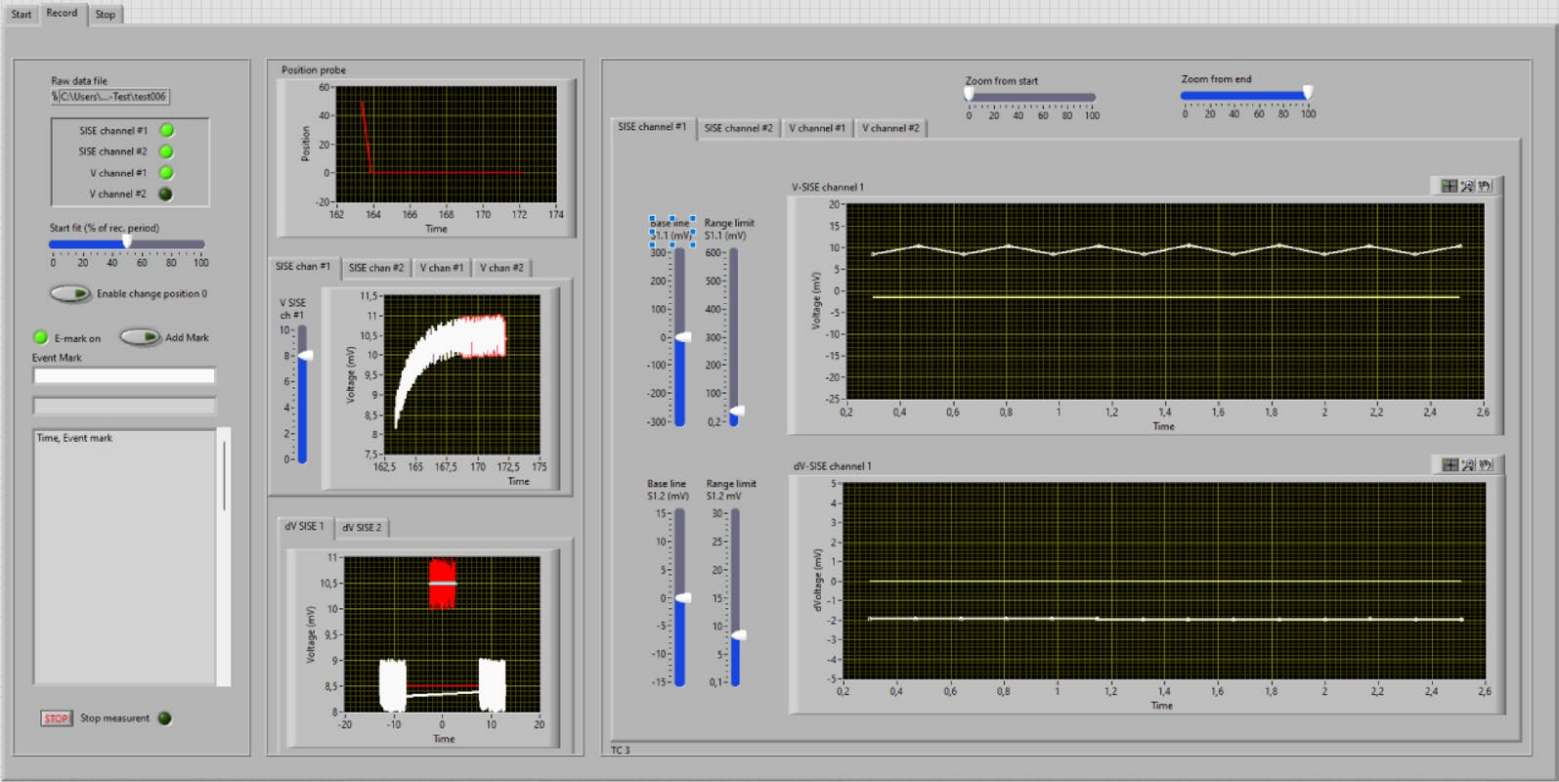
Measurement window of the SISE-Monitor (demo Version). *Windows on left*: Upper window shows overview of the channels that are recorded. The “Start fit” slider allows selection of the period of the measurement that is used for calculation of the voltage difference between position 0 and position 1. The lower windows can be used to enter “event marks” that will be stored in the data file. *Windows in the middle*: Upper graph; the position of the electrode in µm distance from position 0 plotted against time. Middle graph: the voltage signal of the electrode, plotted against time (in the demo version a slider is provided to manipulate the voltage). Lower graph: the voltage difference between position 0 and 1 is determined, by comparing the linear regression of the first and third measurements at one position, with that of the other position in the middle. Only the last part of the voltage recordings (set with the “Start fit” slider) is taken into account. *Windows on right*: Voltage signal (upper graph) and the voltage difference between position 0 and 1 (lower graph) plotted against time (in min.). The base line and voltage range can be changed with sliders on the left, while the slider above the graph can be used to alter the time range

During the measurements, the position of the scanning electrode is registered every 10 ms and displayed in the upper middle graph of the measurement window (Fig. 4). The concurring change in the voltage signal is documented in central graph, while the lower middle graph depicts the procedure to determine the voltage difference between position 0 and position 1.

The voltage signal of the ion-selective electrode is measured with a microelectrode amplifier (VF-102, Bio-Logic, Claix, France, or similar). This amplifier is equipped with a high impedance headstage (input impedance > 10^14^ Ω, HS111, Bio-Logic, or similar), while a custom-made low pass filter, which amplifies the signal − 33 times, is mounted to the output (see Test Protocol, Fig. S4). The minimal noise level of this system is 30 µV, using the NI-USB-6259 A/D converter. However, depending on the properties of the ion-selective electrode, it is likely that the noise level in normal SISE-recordings will be considerably higher.

Voltage data acquired by the amplifier are plotted against time in the central graph of the measurement window (Fig. 4). Due to the slow exponential response of ion-selective electrodes, the change in voltage signal will be delayed compared to movement of the electrode. By default, the voltage of the electrode is assumed to become stable after 5 s, this period is indicated by a “red shadow” behind the white curve, and these data points are used to calculate the average voltage values and voltage difference between position 0 and position 1. During the measurements, the experimenter can adjust the period for which data are averaged with the “start fit” slider. Note that the position of the “start fit” slider does not affect the stored data, and the period for which the data are averaged thus can be altered at a later stage in the SISE-Analyser.

Because of the tendency of ion-selective electrodes to slowly drift with respect to their output voltage, a procedure was developed Newman (2001) to correct for this phenomenon. This procedure is depicted in the lower middle graph of the measurement window (Fig. 4). It shows the selected data points (starting at the time point chosen with the start fit slider) of the last three measurements. The first and last measurements are at the same position (position 0 in Fig. 4), while data in the middle were collected at the other position. Next, a regression line is obtained for the data points of the first and last measurements, followed by calculation of the regression line through datapoints of the middle measurement, using the same angle as found for the first regression line. The dV (Vposition0-VPosition1) value can be deduced from the distance along the Y-Axis between both regression lines (2 mV in Fig. 4). These dV values are plotted against time in the right window, lower graph (Fig. 4) and indicate the changes in ion fluxes in time. 

### Data storage

Data are stored in an American Standard Code for Information Interchange (ASCII)-text file during each cycle of the measurement loop (approximately 10 ms). Comments to the experiment can be entered in the “Event Mark” window and will be stored in the “Event Mark” list and datafile, after clicking the “Add Mark” button. This list will also show if the position of the manipulator is changed during the measurements, which can be enabled by activating the “Enable Change Position” switch (Fig. 4, left panel). The measurement is stopped by pushing the “stop button” at the bottom of the left window (Fig. 4). Once the “stop button” is activated, the last position loop will be completed, followed by the activation of the “termination module” (Fig. 1). In the termination module the data file is closed, and a new measurement can be started by selecting “New measurement”, or the application can be stopped with “Stop program”. Most of the steps described above can also be performed in the demo version of the SISE-monitor and the “synthetic data” can be stored and analysed in the SISE-Analyser applications.

### SISE-analyser

#### Loading and scrolling data

**Fig. 5:**
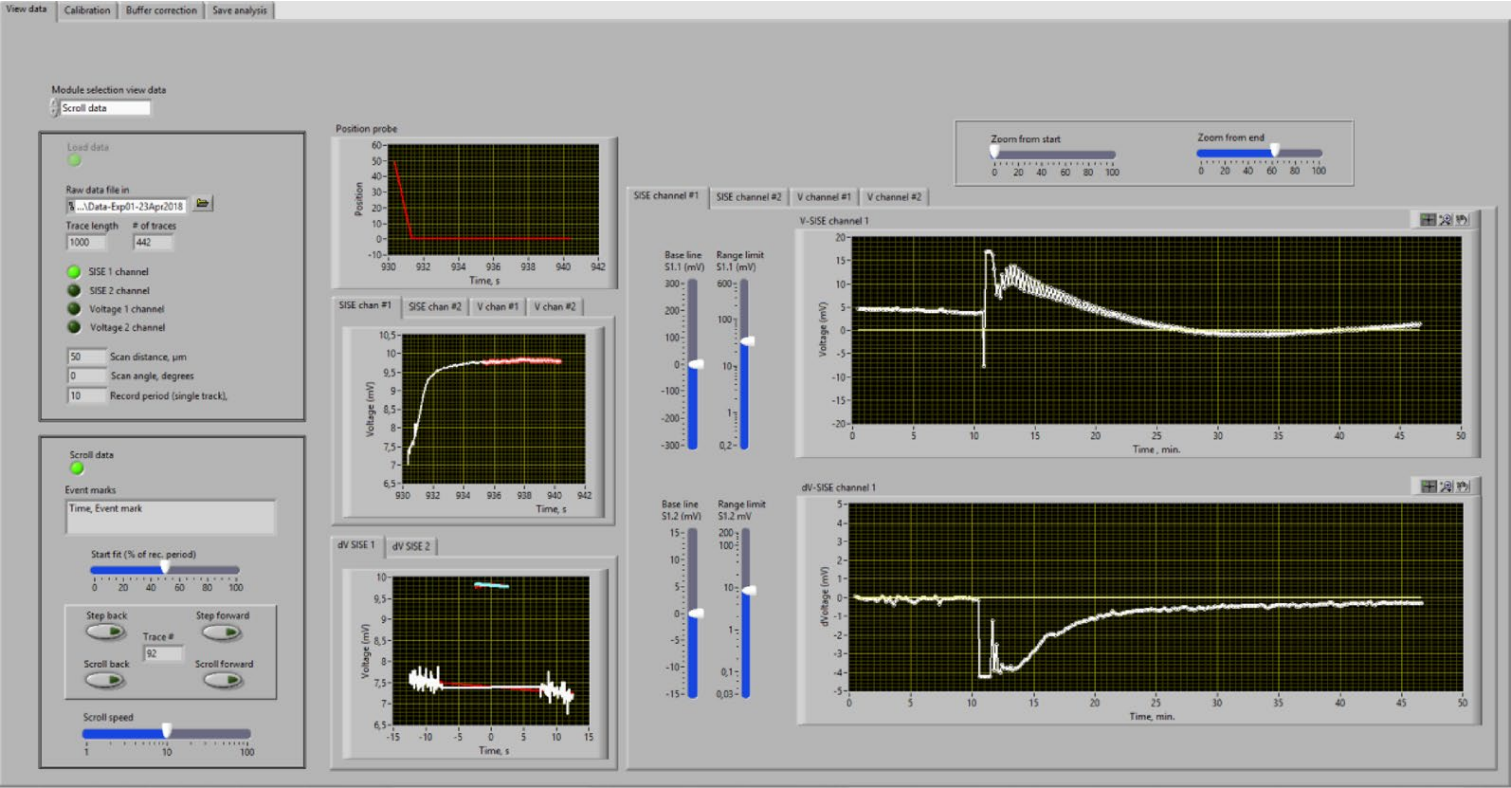
View data window of the SISE-Analyser. *Windows on left*: Sub-modules within the “View data window” can be activated with the selector in upper left corner. A datafile is uploaded and parameters of the measurement are displayed in the “load data” module. The data can be viewed in the “Scroll data” window, in which “step back” and “step forward” enable to switch between measurements and the “Scroll back” and “Scroll forward” allow rapid scrolling of the data. *Windows in the middle*: Upper graph; the position of the electrode in µm distance from position 0, plotted against time (s). Middle graph; the voltage signal of the electrode in time. The measurement that is shown, was conducted with a K^+^ selective-electrode close to a barley root, in a bath solution with 0.5 mM KCl and 0.1 mM CaCl_2_. After 10 min. NaCl was added to a concentration of 100 mM [22]. Lower graph; the voltage difference between position 0 and 1, determined by comparing the linear regression of two measurements at one position with that of the other position. Only part of the voltage recording is used, indicated by the “red shadow” in the central graph (set with “Start fit” slider). *Windows on right*: Voltage signal (upper graph) and the voltage difference between position 0 and 1 (lower graph). The base line and voltage range can be changed with the sliders on the left, while the sliders above the graphs can be used to alter the time range

Data obtained with the SISE-Monitor can be viewed with the SISE-analyser and converted into ion concentrations and ion-flux rates. In the first step, a data file is re-analysed using the same procedures as in the SISE-Monitor. To this purpose, scroll switches can be used to view each measurement and recalculate the average voltage data (V) and voltage difference signals (dV) (Fig. 5). If all measurements have been covered, the virtual LED next to the scroll switches will turn green and allow the determination of ion concentrations and fluxes.

In Fig. 5 the analysis of a K^+^-flux measurement on a root of a barley seedling is shown. At the start of the experiment, the lack of K^+^ fluxes is evident from dV values close to zero (Fig. 5, lower right graph). After 10 min. NaCl was added to a concentration of 100 mM, which first caused convection and rapid changes in V and dV (Fig. 5 right graphs). After 13 min the voltage signal became stable and showed regular changes between position 0 and 1 (Fig. 5 upper right graph) and a change of dV to negative values, which indicates the root cells extruded K^+^ (Fig. 5 lower right graph), as previously reported by Chen et al. (2005) [22] .

### Calculation of ion fluxes

**Fig. 6:**
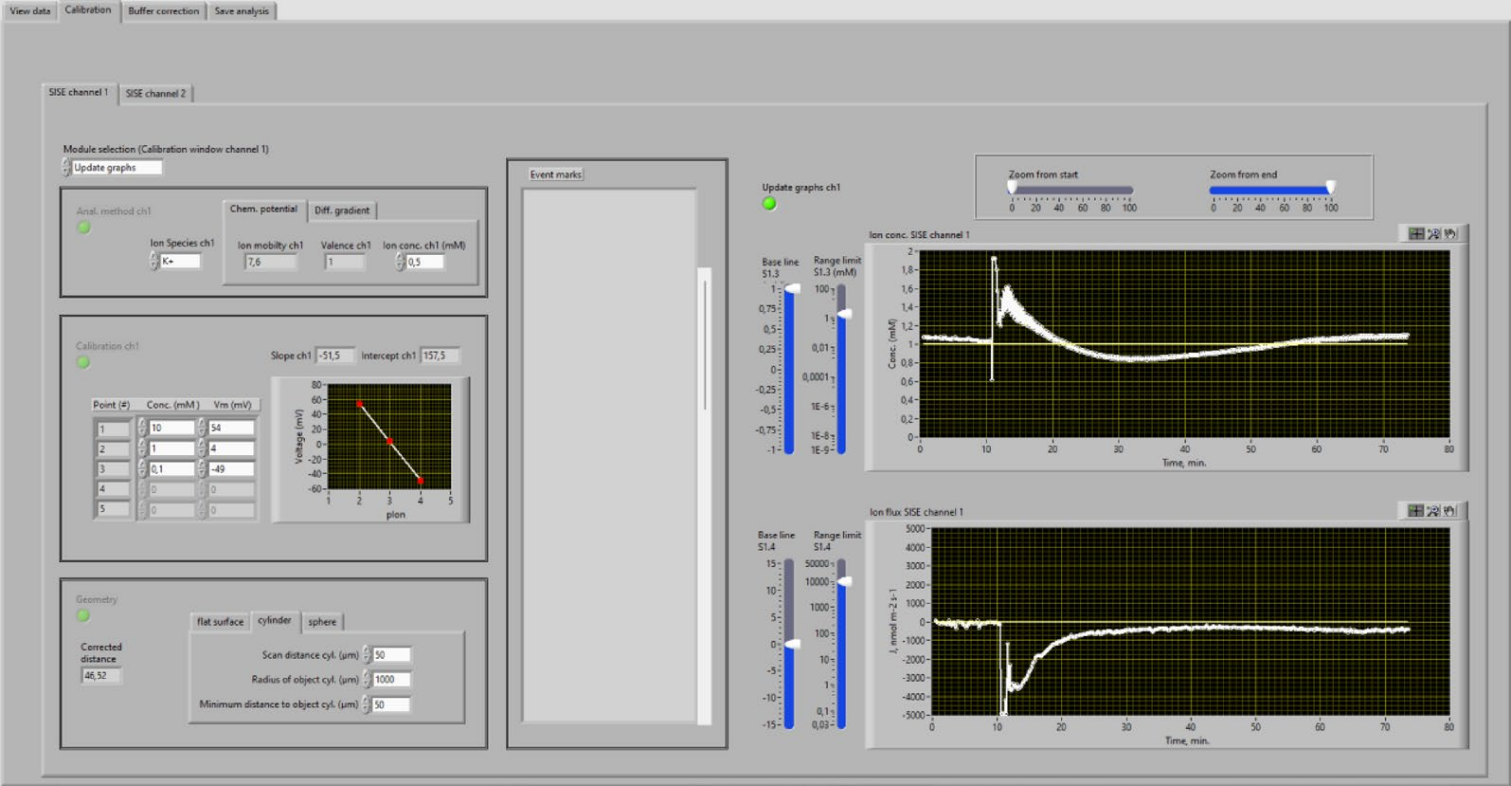
Calibration window of the SISE-analyser. *Windows on left*: Sub-modules within the “Calibration window” can be activated with the selector in upper left corner. In the “Analysis method” sub-module either the “chemical potential” or “diffusion gradient” options can be selected. Upon selection of the “chemical potential” the ion species and its concentration needs to be entered, while the diffusion gradient only requires the ion species selection. Calibration values of the ion-selective electrode are entered in the table of the “calibration” window and are shown in a graph. The size and shape of the tissue, or cell, needs to be provided in the “Geometry module”, in which a corrected distance is calculated for objects with a cylindrical, or spherical shape. *Middle window*: The “event marks” that were entered during the experiment will be shown in the “Event marks” list. *Windows on right*: Ion concentration in mM (upper graph) and the calculated ion flux (K^+^ flux in this experiment) (lower graph) plotted against time (in min.). The base line and voltage range can be changed with sliders on the left, while the sliders above the graphs can be used to alter the time range

The SISE-Analyser110 enables the calculation of ion fluxes, either by using the chemical potential, as described by Newman, 2001 [13], or based on an ion concentration gradient, as conducted by Smith et al., 1994 [15]. For both approaches the voltage values at position 0 and position 1 are used, which are obtained with linear fits through the data points before, during and after the measurement of interest. This procedure is identical as described for the SISE-Monitor above and shown in lower middle graph of Fig. 5. Once the voltage values of all measurements have been obtained, the ion fluxes can be determined in the “calibration” module (Fig. 6).

Four submodules must be executed in the “calibration” module, to calculate the ion fluxes (Fig. 6). In the “analyses method” sub-module (Fig. 6 upper left window), either the chemical potential, or diffusion gradient can be chosen. If the chemical potential is used, the ion species needs to be selected and its average concentration in the bath should be entered. Based on these values and the voltage difference (dV) between both positions, ion fluxes are calculated with Eqs. 1,

1 $$\:\text{J}=C\:u\:\left(\frac{58/z}{{slope}_{cal.\:curve}}\:\right)\left(\frac{dV}{dx}\:\right)$$

in which J is the ion flux (in mol m^-2^ s^-1^), C is the average concentration of the ion in the bath solution (in mol m^-3^), u is the mobility of the ion (in m^2^ V^-1^ s^-1^), 58/z the slope of an ideally ion-selective electrode (in which z is the valency of the ion), slope_cal.curve_ is the actual slope of the calibration curve, dV, the voltage difference between position 0 and position 1 and dx the corrected distance of movement of the electrode.

Note that the ion mobility u in Eq. 1 is given in m^2^ V^-1^ s^-1^, which is more common as the value of m s^-1^/(J mol^-1^) used by Newman (2001). This procedure thus uses dV to calculate the ion flux, using only the slope of the calibration curve and not its intercept with the Y-axis. As a result, the procedure is not sensitive to drift of the voltage signal of the ion selective electrode, as long as the slope of the curve is not affected. However, the concentration difference between Position 1 (at the largest distance of the studied cells) and the bulk solution is assumed to be neglectable. This assumption may not hold at large ion fluxes, such as the K^+^ efflux from barley roots induced by NaCl (Fig. 6, upper right graph).

Alternatively, the “Diffusion gradient” method can be chosen in the “Analysis method” submodule. With this approach the ion concentrations are calculated for position 0 and 1, using the same voltage values as for the “chemical potential” method. These voltage values are used to determine the difference in ion concentration at both positions. Subsequently, the ion flux is calculated with Fick’s law of diffusion,

2 $$\:J\:=D\:\frac{\text{d}\text{C}}{\text{d}\text{x}}$$

, in which D is the diffusion coefficient of the ion and dC the concentration difference between position 0 and 1 and dx the distance between both positions.

**Fig. 7:**
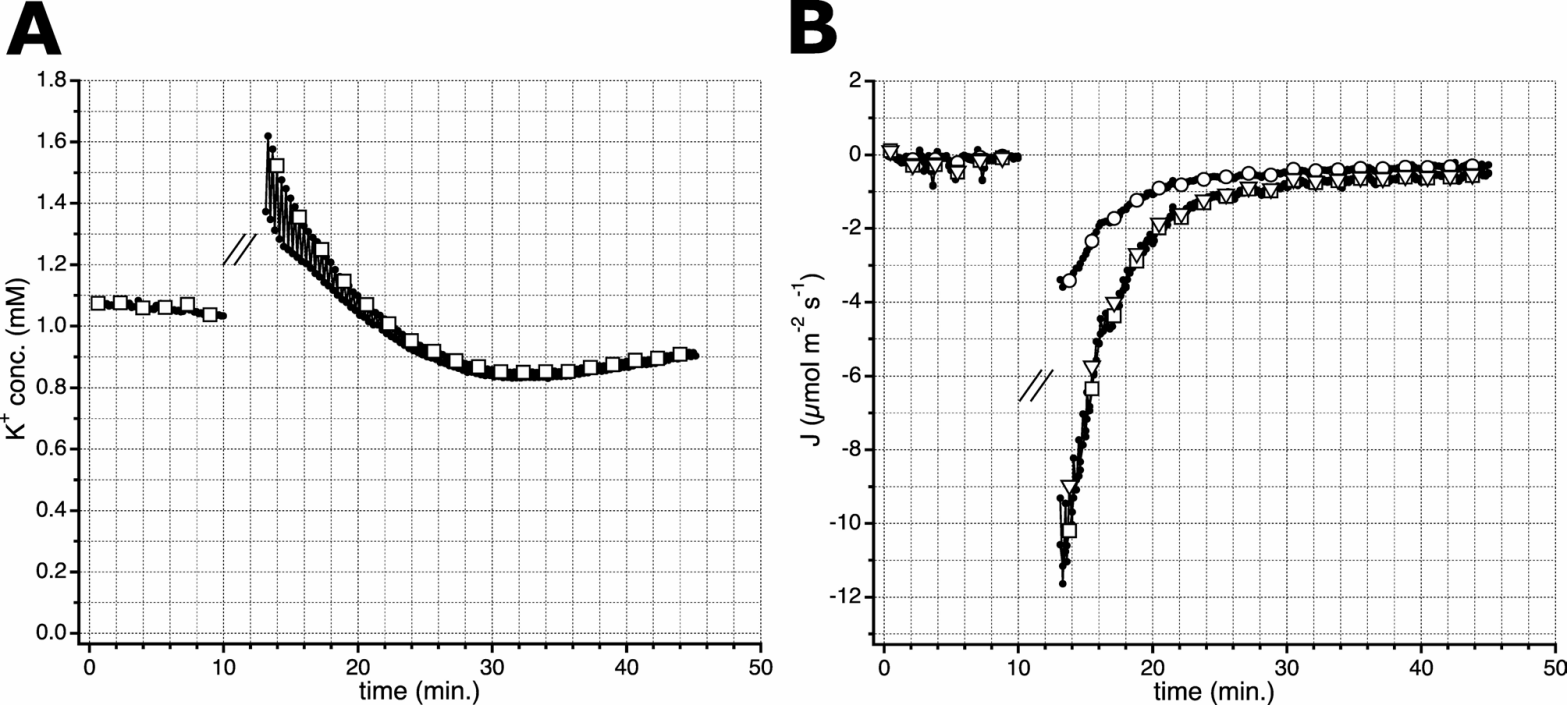
Changes in K^+^ concentration and fluxes at a barley root, elicited by 100 mM NaCl. **A** Alternating K^+^ concentration measurements at 50 μm (Position 0), or 100 μm (Position 1) from the root epidermis, plotted against time. After 10 min. NaCl solution was added to a concentration of 100 mM. **B** Ion flux data determined with the “chemical potential method” (open circles) and “diffusion gradient approach” (open squares) plotted against time, for clarity only symbols are shown for every 10th datapoint). The open triangles show K^+^-fluxes obtained with the “chemical potential method” that were corrected for the difference in K^+^ concentration measured near the root and in the bulk solution. Note that these data overlap with that determined with the diffusion gradient. The barley root was stimulated by adding NaCl to a concentration of 100 mM after 10 min. Note that the traces in A and B are disrupted from 10 to 13 min, as the addition of NaCl solution caused convection, and thus distorted the SISE-measurement

 A comparison of K^+^ concentration and K^+^ fluxes calculated with the “chemical potential” and “diffusion gradient” methods is shown in Fig. 7. Addition of 100 mM NaCl after 10 min. caused a transient increase of the K^+^ concentration to approximately 1.5 mM (Fig. 6 upper right graph, Fig. 7A). During this period the K^+^-flux reached a value of approximately 3000 nmol m^-2^ s^-1^, when calculated with Eq. 1 (Fig. 6 lower right graph, Fig. 7B). This K^+^-flux is within the range of values reported by Chen et al. (2005). Based on the diffusion gradient, a K^+^-flux is determined that is 3 times higher (Fig. 7B). However, if the values obtained with the “chemical potential” are corrected for the difference in K^+^ concentration at position 1 and the bulk solution, an overlap is found for the K^+^ flux values calculated with both approaches (Fig. 7B).

Calculation of the ion flux values requires a calibration curve of the ion selective electrode (left window, Fig. 6). To this purpose, values must be entered for the ion concentrations in the calibration solutions (in mM) together with corresponding voltage signals (in mV). As soon as these values are entered, a calibration graph will appear in the window of this sub-module and the virtual LED will turn green

 In case that the measured tissue can be regarded as a flat surface, only the distance between position 0 and position 1 needs to be entered (in µm), in the “Geometry” sub-module. Corrections for cylindrically shaped tissues, or spheres, are made as described by Newman (2001). The “Corrected distance” of movement is shown on the left side of the “Geometry” window. Once all information of the sub-modules (left windows in Fig. 5) is available (virtual LEDs turn green), the “update graphs” window can be selected and ion concentrations are calculated (Fig. 6, right window, upper graph)

### Buffer correction

Ion flux data that are determined in the “Calibration” module resemble the flux of free ions. However, if the ion is buffered in the solution, it will form a complex with the buffer molecules ([HA]) and cause a gradient of conjugated buffer molecules. In other words, part of the H^+^ flux across the plasma membrane will cause a flux of conjugated buffer, instead of a flux of free H^+^. For this reason, a correction procedure for pH buffers has been developed by Arif et al. (1995) [23], which is integrated in the SISE-analyser “Buffer correction” module. Within this module, common buffers such as 2-(N-morpholino) ethanesulfonic acid (Mes) and tris(hydroxymethyl)aminomethane (TRIS) can be selected (for mobility see supplementary information part 3). In addition, some organic acid buffers are provided but note that these buffers can have several pK_A_ values and the values only can only be used if the pH of the solution is close (less than 0.25 pH units difference) to the pK_A_ value that is indicated. Additional buffers can be introduced in the VI-version of SISE-analyser using the community edition of LabView.

Just as for the ion flux calculations, two procedures are provided to correct the ion flux values for the flux of the protonated buffer. In case that the “Chemical potential” is used in the “Calibration” module, the flux of protonated buffer will be determined with the following equation derived by Arif et al. (1995):

3 $$\:{\text{r}=\frac{\text{u}\text{H}\text{A}}{\text{u}{\text{H}}^{+}}\:C\:{10}^{-3}\:{10}^{pK}\:\left(\frac{{10}^{pH}}{{10}^{pK}+\:{10}^{pH}}\right)}^{2}$$

,where r is the ratio between the flux of H^+^ and the protonated buffer (HA), u_HA_ the mobility of the protonated buffer, u_H+_ the mobility of H^+^ and C is the total concentration of the buffer ([AH] + [A^−^]). The total flux is calculated as: J_total_ = (*r* + 1) J_H+_.

If the ion fluxes are calculated with Fick’s law, the concentration of protonated buffer ([HA]) is calculated with the following equation:

4 $$\:\left[HA\right]=\:C\:\frac{{10}^{pK}}{{10}^{pH}+\:{10}^{pK}}$$

The difference in concentration at Position 0 and 1 is used to determine the flux of protonated buffer with Fick’s law (Eq. 1) and this value is added to the flux of free H^+^ ions.

### Data storage

Storage of the analysis occurs in the “save analysis” module, in which the “analysis output file” is entered and data can be selected, which will be written in a text file. Depending on the number of channels that were activated during the measurement, and the type of analysis (with, or without buffer correction), the “analysis out file” can have up to 15 columns of data.

## Discussion/Conclusion

The SISE-programs that are provided on GitHub are freely available and open source. This ensures that the routines used to sample data and calculate ion fluxes can be evaluated by the users and modified upon demand. Version 110 of the SISE-Analyser comes with two options to calculate ion fluxes, the default method is based on chemical potential of the ion of interest, as described by Newman (2001). Alternatively, an approach can be chosen to determine the ion flux with Fick’s law, as developed by Smith et al. (1994).

The method based on the “chemical potential” has the advantage that it uses only the slope of the electrode calibration curve, and not the intercept with the Y-Axes. This makes the procedure less sensitive to drift of the electrode’s voltage signal during the experiments, as compared to the method based on the “concentration gradient”. However, the “chemical potential” method assumes only a neglectable difference in ion concentration of the bulk solution and that at position 1 (the position at largest distance from the studied cell, or tissue). In case of a large ion concentration gradients, this assumption will not hold, as shown in Fig. 7B. The approach based on the chemical potential underestimates the ion flux, which can be corrected by using the measured ion concentration at position 1, instead of assuming the same ion concentration as in the bulk bath solution.

Apparently, the approaches of Smith et al. (1994) and Newman (2001) will give the same values, as long as the concentration of position 1 is close to the average value in the bath solution. Because of its relative low sensitivity to electrode drift, the method of Newman is especially useful for small ion fluxes. At larger ion fluxes, the ion concentration has to be corrected for the changes at Position 1, or one can use Fick’s law of diffusion.

According to the Nernst-Planck equation, ion fluxes depend on diffusion and electromigration [24]. As explained by Newman (2001), an ion selective electrode converts the diffusion gradient into an electrical potential that can be added to the force of the electrical field on the ion of interest. The “chemical potential” method thus considers both diffusion and electromigration, whereas the “diffusion gradient” approach is only based on diffusion.

Despite of the ability of the “chemical potential” method to consider the impact of electrical fields, care should be taken to interpret ion flux measurements under such conditions. An electrical field will affect the movement of all ions in the solution and as a result the flux of the ion species of interest must not necessarily represent the flux of this ion species across the plasma membrane. In other words, part of the ion current across the plasma membrane may be compensated by fluxes of other ion species in the bath solution. This topic requires further examination, as ion flux measurements will be very useful to study transport proteins in heterologous expression systems, such as *Xenopus laevis* oocytes. If these cells are studied with the two-electrode voltage-clamp system, electrical fields in the bath solution will be unavoidable and their impact on ion-fluxes needs to be considered.

There are several features that we intend to introduce into future versions of the SISE-programs. One of these features is to capture images that document the position of the electrode, relative to the object that is studied. This option was already implemented by Shipley and Feijo (1999) [16] and is especially useful, if ion fluxes are determined at range of positions along the surface of a tissue. Such an experimental approach has provided data of ion-uptake gradients, along pollen tubes [7, 11, 25], or roots [26, 27]. Image capturing and analysis thus will be a valuable extension planned to be included in future versions of the SISE-applications.

Camera control and image analysis can be conducted with “Micromanager”, a free software package that enables simultaneous control of microscope cameras and illumination devices [28]. The combined use of Micromanager and the SISE-Monitor will enable imaging and ion-flux measurements simultaneously. Moreover, the Micromanager can be used to conduct measurements with genetically encoded fluorescent ion-indicators. The simultaneous use of Micromanager and the SISE-Monitor application thus may enable linking extracellular ion fluxes to changes in the cytosolic ion concentration of plant cells.

Micromanager uses the ImageJ application [29] to visualize and analyse the images that are captured. This program thus will also be useful to analyse the captured images, in combination with ion flux analysis that is carried out with the SISE-Analyser. We intend to synchronize the ion flux- and image data-analysis and thus enable a rapid and easy procedure to link the results of both methods.

Because of the availability of the source code in the virtual instrument-version of the SISE-programs, it can be modified and expanded by any of the users. We would welcome, if such new attributes of the SISE-programs would be made publicly available. This could provide a range of versions of the SISE-programs, with a variety of helpful features that would enable SISE-users to find an optimal solution for their needs.

## Supplementary Information

Below is the link to the electronic supplementary material.

Supplementary Material 1.

## Data Availability

The software will be available on GitHub, as *.exe and *.vi files.

## Abbreviations

AI channel: Analog Input channel
ASCII: American Standard Code for Information Interchange
A/D converter: Analog-to-digital converter
MIFE: Microelectrode Ion-Flux Estimation
NI: National Instruments Corp
NMT: Non-invasive Micro-test system
SIET: Scanning Ion-selective Electrode Technique
SISE: Scanning Ion-Selective Electrode
VISA: Virtual Instrument Software Architecture
*.exe: Windows executable file
*.vi: LabView virtual instrument file
